# Shizukaol D, a Dimeric Sesquiterpene Isolated from *Chloranthus serratus*, Represses the Growth of Human Liver Cancer Cells by Modulating Wnt Signalling Pathway

**DOI:** 10.1371/journal.pone.0152012

**Published:** 2016-03-24

**Authors:** Lisha Tang, Hengrui Zhu, Xianmei Yang, Fang Xie, Jingtao Peng, Deke Jiang, Jun Xie, Meiyan Qi, Long Yu

**Affiliations:** 1 State Key Laboratory of Genetic Engineering, School of Life Sciences, Fudan University, Shanghai, China; 2 The Wistar Institute, Philadelphia, Pennsylvania, United States of America; 3 Mayo Clinic, Rochester, Minnesota, 55905, United States of America; 4 Department of Urology, Shanghai First People’s Hospital, Shanghai Jiaotong University, Shanghai, China; 5 Program for Personalized Cancer Care, NorthShore University HealthSystem, Pritzker School of Medicine, University of Chicago, Illinois, United States of America; 6 The Institute for Nutritional Sciences, Shanghai Institutes for Biological Sciences, Chinese Academy of Sciences, Shanghai, China; University of Kentucky, UNITED STATES

## Abstract

Natural products have become sources of developing new drugs for the treatment of cancer. To seek candidate compounds that inhibit the growth of liver cancer, components of *Chloranthus serratus* were tested. Here, we report that shizukaol D, a dimeric sesquiterpene from *Chloranthus serratus*, exerted a growth inhibition effect on liver cancer cells in a dose- and time-dependent manner. We demonstrated that shizukaol D induced cells to undergo apoptosis. More importantly, shizukaol D attenuated Wnt signalling and reduced the expression of endogenous Wnt target genes, which resulted in decreased expression of β-catenin. Collectively, this study demonstrated that shizukaol D inhibited the growth of liver cancer cells by modulating Wnt pathway.

## Introduction

Liver cancer is the third leading cause of cancer-related death worldwide [1]. In patients with liver cancer, surgical treatments offer a high rate of complete response and offer a potential for cure [2]. However, tumour resectability can be limited by tumour extent, location, and underlying liver dysfunction at late stages of the disease; as such, resection is only feasible in a minority of patients [3]. To date, in patients with advanced hepatocellular carcinoma (HCC), only sorafenib has been shown to increase overall rates of survival efficiently [4]. Even in the United States, the incidence of hepatocellular carcinoma has tripled, whereas the 5-year survival rate has remained below 12% [5]. Thus, alternative strategies for treating this aggressive tumour type are urgently needed.

In recent years, an increasing number of investigators have become interested in screening natural products for potential anti-cancer agents, including Chinese Medical Herbs (CMH). *Chloranthus serratus*, which belongs to the family *Chloranthaceae*, is a perennial herb that is primarily distributed throughout northern China, Korea and Japan. Because of its reputation in facilitating blood circulation and dispersing blood stasis, a series of compounds have been isolated from *Chloranthus serratus*, including threo-1-(1-Methoxy-2-hydroxypropyl)-2-methoxy-4,5-methylenedioxybenzene and erythro-1-(1-methoxy-2-hydroxypropyl)-2-methoxy-4,5-methylenedioxybenzene [6], serralabdanes A–E [7] and serratustones A -B [8]. Lindenane-type sesquiterpenoids are recognized as the characteristic taxonomic symbol of the *Chloranthaceae* family. Many of these sesquiterpenoids exhibit favourable anti-inflammatory bioactivities. For example, chloramultilide B possesses inhibitory activities against *Candida albicans* and *Candida parapsilosis* with MIC values equal to 0.068 μmol/L [9]. In addition to evaluating their anti-inflammatory effects, more studies have focused on the activities of sesquiterpenoids on cancer cells. The dimeric sesquiterpenoid cycloshizukaol A has been shown to markedly inhibit ICAM-1 expression in HL-60 cells in a dose-dependent manner [10], and antrocin, a sesquiterpene lactone, induces apoptotic cell death of human bladder cancer 5637 cell lines via both extrinsic and intrinsic signaling pathways[11]. The growth inhibitory effects of xanthorrhizol, a sesquiterpenoid from the rhizome of *Curcuma xanthorrhiza*, against human colon cancer HCT116 cells, are related to the cell cycle arrest and induction of apoptosis [12].

**Fig 1:**
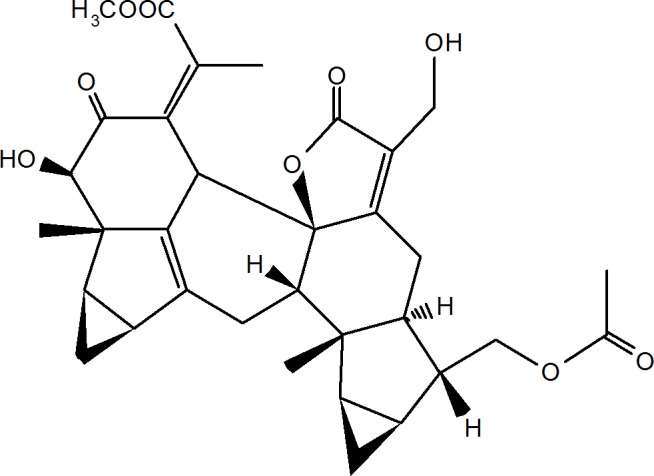
Structure of shizukaol D.

Shizukaols are a series of typical sesquiterpenoid dimer derivatives. Shizukaol A was first isolated from *Chloranthus japonicus* in 1990 [13]. Shizukaol D (as shown in Fig 1) has been isolated from *Chloranthus serratus* [14]. Previous studies on the bioactivity of shizukaol D are extremely limited and have primarily focused on its anti-inflammatory activities [15]. It has also been shown to inhibit AMPK-dependent lipid content in hepatic cells [16] and to increase glucose consumption in L6 cells [17].

In this study, various isolations from *Chloranthus serratus* were tested for their effects on cancer cells. From these isolations, shizukaol D was found to induce growth inhibition and attenuate Wingless-Int (Wnt) pathway signalling in liver cancer cells.

## Materials and Methods

### Chemicals and plasmids

Shizukaol D (Fig 1) was isolated from *Chloranthus serratus* according to a previously published method [14] by Bio Bli Ltd.com. Following this, the compound was prepared as a 100mmol/L stock in dimethyl sulfoxide (DMSO) and stored at 4°C. The primary antibodies that were used in western blotting included antibodies for PARP (Cell Signalling), LRP (Cell Signalling), p-LRP (Cell Signalling), Dvl2 (Cell Signalling), Axin2 (Cell Signalling), β-catenin (BD), GSK-3β (Cell Signalling), p-GSK-3β (Cell Signalling), β-actin (Sigma) and GAPDH (Abmart). A wild type β-catenin plasmid (wt-β-catenin) was prepared by inserting a gene encoding β-catenin into a pcDNA3.0 plasmid, whereas a mutant β-catenin plasmid (mut-β-catenin) was prepared by inserting a gene encoding β-catenin with mutations at S33A, S37A and T41A into pcDNA3.0.

### Cell lines and cell culture

The human cancer cell lines SMMC-7721, SK-HEP1 and HepG2, were obtained from the American Type Culture Collection (ATCC). Additional cell lines, including Focus, HEK-293T, L Wnt-3A and QGY-7703, were purchased from the Institute of Cell Library of China. SMMC-7721, Focus, and HepG2 cells were cultured in Dulbecco’s Modified Eagle’s Medium (DMEM, Invitogen) supplemented with 10% foetal bovine serum (FBS, Gibico), and SK-HEP1 and QGY-7703 cells were cultured in RPMI-1640 medium (Invitogen) supplemented with 10% FBS. L Wnt-3Awas cultured in DMEM with G418 to yield wnt3a conditioned medium. All of the cells were cultured at 37°Cin a humidified incubator with 5% CO_2_.

### CCK-8 assay

Cell responses to shizukaol D were assessed using 2-(2-methoxy-4-nitrophenyl)-3-(4-nitrophenyl)-5-(2,4-disulfophenyl)-2H-tetrazolium monosodium salt in a modified CCK-8 cellular proliferation assay kit (Roche Diagnostics, IN). Cells were plated into 96-well plates and then exposed to a range of shizukaol D concentrations for varying lengths of time. The culture media was removed before adding 90 μL of fresh media (without FBS) and 10 μL Cell Counting Kit-8 solution to each well. The plates were incubated for an additional 2 hours at 37°C, after which absorbance was measured at 450 nm using a microplate reader (model 550, Bio-Rad, CA). The percentage of inhibition relative to an untreated control is illustrated. Each experiment was performed at least three times independently.

### Evaluation of sub-G1 cells

Focus cells were planted in 6-well plates and incubated in DMEM with 0, 12.50, 25.00 or 50.00 μmol/L of Shizukaol D for 48 hours. DMEM with 1.00% DMSO was used as a control. The cells were fixed and stained in phosphate-buffered saline (PBS, 140 mmol/L NaCl, 2.7 mmol/L KCl, 10 mmol/L Na_2_HPO_4_ and 1.8 mmol/L KH_2_PO_4_, PH = 7.4) containing 50 μg/mL propidium iodide and 0.03% Triton X-100 before being analysed by flow cytometry (FCM, FAC Star Plus, Mod-Fit LT V2.0; Becton Dickinson, Franklin Lakes, NJ).

### Colony formation assay

Cells were treated with 3.13, 6.25, 12.50 or 25.00 μmol/L Shizukaol D for 48 hours before being plated into 6-well plates at a density of 500 cells per well and cultured in normal media for 7–10 days until colonies formed that contained more than 50 cells. A solution of 0.1% DMSO was set as a control. After fixation with 4% polymethanol for 10 minutes, the colonies were stained with 1.0% crystal violet for 30 minutes.

### Western blot analysis

Cells were lysed in Cell Lysis buffer (Cell Signaling). After centrifugation, supernatants were harvested, and total protein was subjected to 10% SDS-PAGE and transferred onto a nitrocellulose membrane (GE). The membrane was blocked with 5% skim milk for 1 h, incubated overnight at 4°C with primary antibodies, and then incubated with secondary antibodies. Antibody binding was detected using enhanced chemiluminescence (GE). The membrane was stained with Ponceau S (Sigma) and probed with β-actin or GAPDH antibody to confirm equivalent loading and protein transfer.

### Immunofluorescence staining

Cells in culture were fixed in 4% paraformaldehyde for 10 minutes. Following this, the cells were treated with 0.2% Triton X-100 in PBS for 20 minutes. The cells were further incubated overnight at 4°C with antibodies specific to β-catenin (BD) and washed with PBS three times before incubated for 2 hours at room temperature with secondary antibodies. After washing with PBS, coverslips (which contained the cells) were then mounted in DAPI-containing mounting media (Beyotime, CN) and imaged on a Zeiss confocal microscope.

### Luciferase reporter assay

HEK-293T cells were cultured in media containing 20 mmol/L of LiCl before transfected with the Wnt signalling reporters TOPflash or FOPflash as previously described [18]. Following this, the cells were treated with 6.25 or 12.50 μmol/L shizukaol D for 24 h. Renilla plasmid was included in all the related transfections. The data are represented as normalised TOP/FOP Flash values.

### Quantitative real-timePCR

Total RNA was extracted from cultured cells using Trizol reagent (Invitrogen) and DNA was removed by DNase (Promega). Reverse transcription was performed using SuperscriptII reverse transcriptase (Takara). Quantitative real-time PCR (QPCR) was performed using SYBR Green I staining (Takara) on an iCycleri QTM system (BioRad) according to the manufacturer’s protocols. Gene expression levels were normalized to the internal GAPDH levels. The conditions were as follows: 38 cycles of three-step PCR (95°C for 40 s, 60°C for 50s, and 72°C for 30 s) after initial denaturation (95°C for 5 min).

### Statistics

Data presented as mean ± standard deviation were at least three sets of independent experiments. T-test analysis was used to determine the significance of statistical differences. Differences were calculated by SPSS and considered significant at P<0.05.

## Results

### Growth inhibition of human liver cancer cells by shizukaol D

This study was initiated to test the cytotoxicity of natural products isolated from *Chloranthus serratus* in different types of cancer cells. Shizukaol D (Fig 1) was the most effective compound among those that have been reported. Liquid phase chromatography assay (S1 Fig), MHz assay (S2 Fig) and HPLC (S3 Fig) confirmed the structure of shizukaol D and its purity. A CCK-8 assay was subsequently used to verify the growth inhibition effect of shizukaol D on liver cancer cells, including the Focus, HepG2, QGY-7703, SMMC-7721 and SK-HEP1 cell lines, at various concentrations (0–200 μmol/L). The cells exhibited different sensitivity to shizukaol D (Table 1). As a positive control, the IC_50_ of doxorubicin (Dox) on Focus cells was measured to be 0.23μmol/L, and the IC_50_ of 5-FU (5-Fluorouracil), which was a commonly used agent for malignant digestive tract tumours [19], was measured to be more than 20 μmol/L.

**Table 1:** IC_50_ values of shizukaol D on liver cancer cell lines.

Cell lines	IC_50_(μmol/L)
SMMC-7721	8.82±1.66
SK-HEP1	9.25±0.57
Focus	6.26±0.85
HepG2	>50
QGY-7703	14.17±1.93

**Fig 2:**
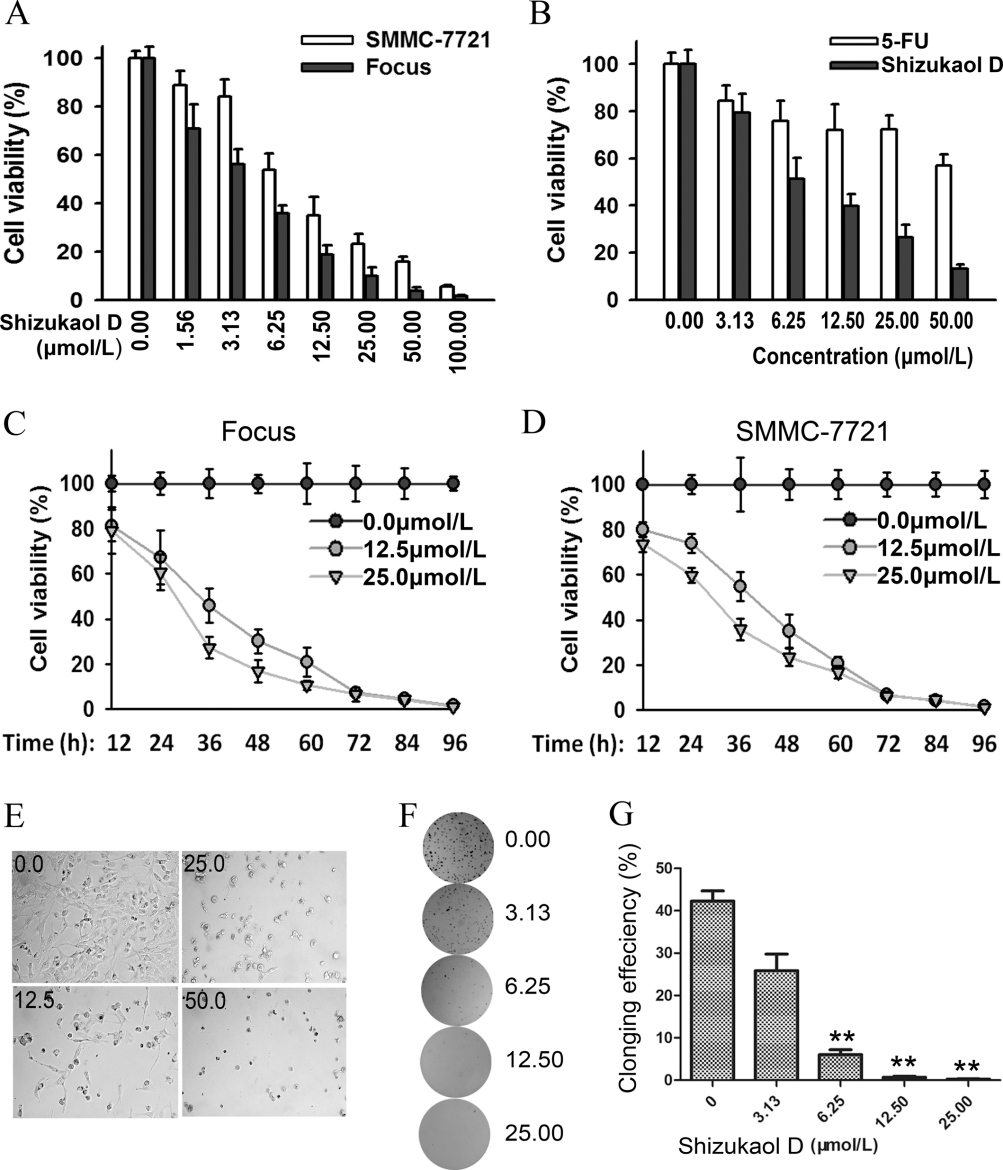
Shizukaol D inhibited the growth of liver cancer cells. (A-D). Cells were seed in a 96 well plate and treated with a series concentration of shizukaol D. Cell proliferation was measured by CCK-8 assay. (A) The viability of Focusand SMMC-7721cells were decreased when the cells were treated with increasing concentration of shizukaol D for 48h.(B) The viability decreased faster when treated with shizukaol D than treated with same concentration of 5-FU in SMMC-7721 cells for 48 h. The cell viability decreased in Focus (C) and SMMC-7721 (D) cells treated by shizukaol D in a dose- and time-dependent manner. (E) The transparent version of Focus cells treated with 0.00, 12.50, 25.00 and 50.00 μmol/L of shizukaol D for 48 hours. (F) Colony formation assay of SMMC-7721 cells. The cells were treated with 0.00, 3.13, 6.25, 12.50 and 25.00 μmol/L of shizukaol D for 48 hours and cells for each concentration were collected respectively, and plated in 6-well plates as 500/well. The cells were cultured with normal medium for 10 to 15 d then. (G) Statistics of cloning efficiency in Figure 2F. **: P<0.01. Values are presented as mean±S.D.

Due to their relative higher sensitivity to shizukaol D, Focus cells and SMMC-7721 were used in the subsequent experiments. We detected the growth inhibitory effects of shizukaol D with increasing concentrations. The proliferation of both cells was largely affected by treatment of shizukaol D at low concentrations, and this effect was more obvious following dose increase (Fig 2A). The growth of Focus cells with shizukaol D treatment also decreased in a time-dependent manner. The cell proliferation exhibited a gradual decrease over a course of 96 h following treatment with shizukaol D with concentrations of 12.50 μmol/L and 25.00 μmol/L, with DMSO as a negative control (Fig 2C). The same trend was also observed in SMMC-7721 cells (Fig 2D). With the increased concentration of shizukaol D, Focus cells became round, floated and even dead in bright field (Fig 2E). Furthermore, colony formation assay indicated that colony formation in SMMC-7721 cells was reduced with exposure to increasing concentrations of shizukaol D (Fig 2F and 2G). Colonies by SMMC-7721 cell were barely detectable when the concentration of shizukaol D reached 12.50 μmol/L, which suggested an impaired proliferation ability of cells with shizukaol D treatment (Fig 2F and 2G). Interestingly, the effect of shizukaol D on SMMC-7721 cells was more than that of 5-FU (5-Fluorouracil), especially when the concentrations of greater than 6.25 μmol/L were used (Fig 2B). In summary, these results indicated that shizukaol D inhibited the growth of liver cancer cells.

### Shizukaol D induced apoptosis in liver cancer cells

**Fig 3:**
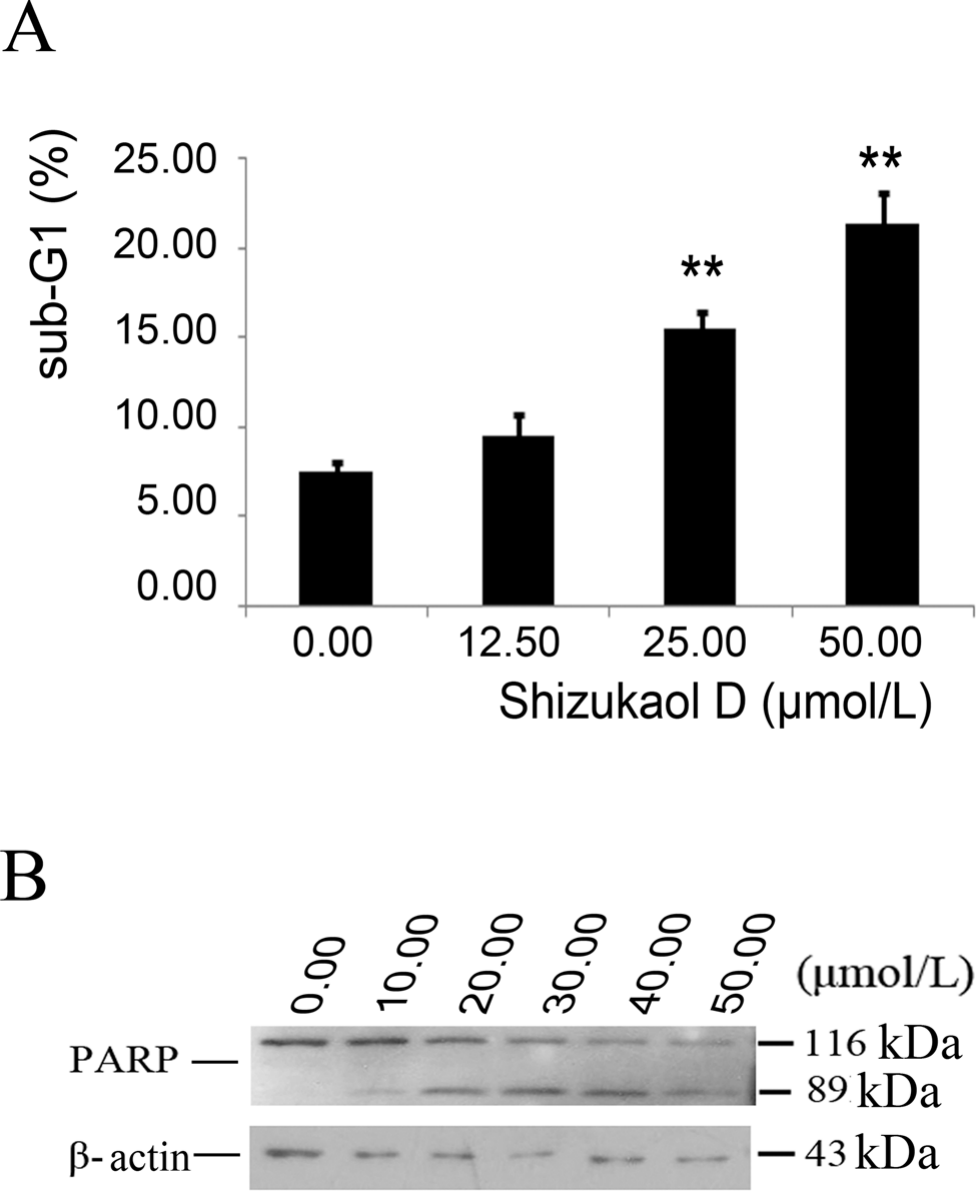
Shizukaol D inducedliver cancer cells to apoptosis. (A) The statistics of the sub-G1 ratio of Focus by FCM. Cells were incubated with shizukaol D for 48 hours before being collected and diluted in PBS with 50μg/ml PI and 0.03% TritonX-100. **: P<0.01. Values are expressed as mean±S.D. (B) Western blot analysis of the expression of PARP. Focus cells were treated with shizukaol D for 48 hbefore collected and then the total protein was subjected to 10% SDS-PAGE and transferred onto the nitrocellulose membrane. β-actin antibody was used to confirm equivalent loading.

Bright-field images of cells showed that shizukaol D induced increased cell death with concentration gradients, which prompted us that shizukaol D could lead to cell apoptosis. We tested several apoptosis markers in liver cancer cells that were treated with shizukaol D at concentrations of 0–50 μmol/L shizukaol D. The results indicated that the sub-G1 ratio of Focus cells that were incubated with shizukaol D increased from 7.49±0.59 to 21.38±1.80 (Fig 3A). Sub-G1 is a peak portion prior to G1 peak in FCM, which implies the formation of apoptotic body. Western blotting confirmed that the cleaved PARP was observed when the concentration of shizukaol D achieved and over 12.50μmol/L, whereas the quantity of pro-PARP decreased (Fig 3B). Thus, the growth inhibitory function of shizukaol D was mediated via inducing apoptosis.

### Shizukaol D attenuated the Wnt pathway

**Fig 4:**
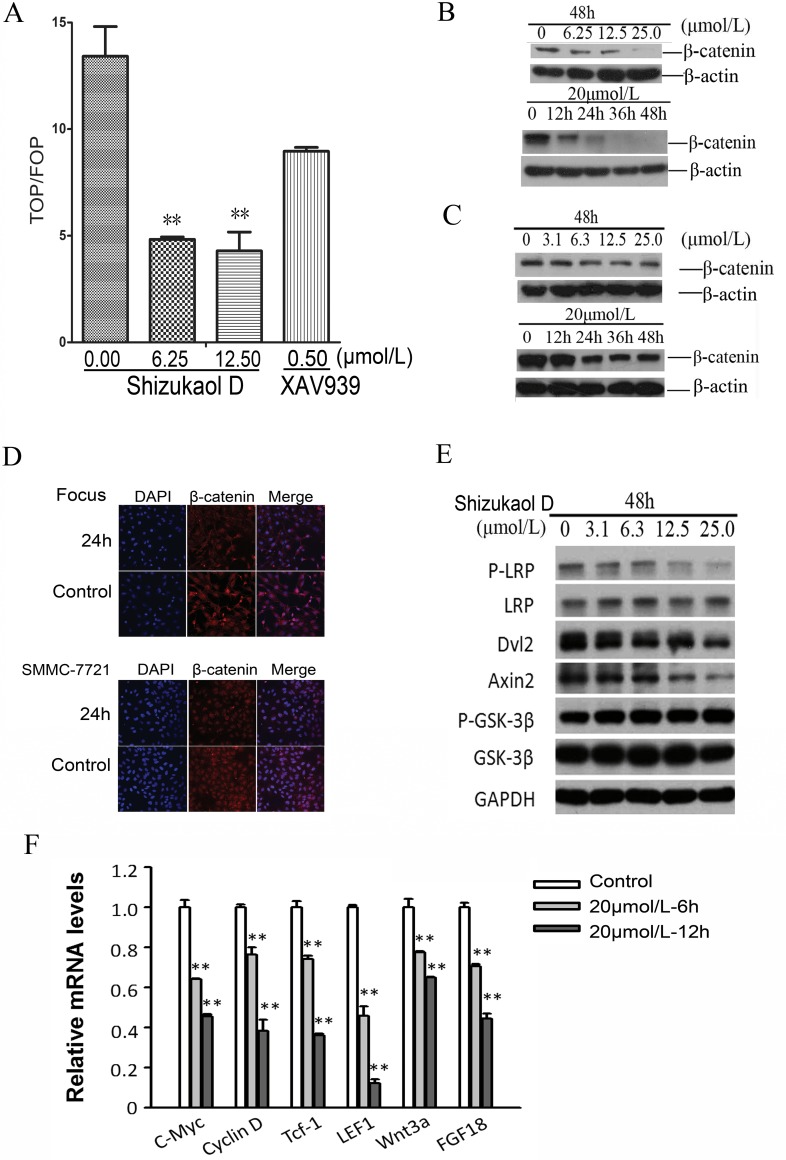
Modulation of Wnt pathway of liver cancer cells by shizukaol D. (A) Shizukaol D decreased TOPflash activity and attenuated TOPflash activity in HEK-293T cells cultured in RPMI-1640 medium containing 50mmol/L of LiCl. Renilla plasmid was included in all the transfections as control. (B-C) Western blot analysis of expression of β-catenin expression in Focus (B) and SMMC-7721 (C) cells treated with shizukaol D. (D) Immunofluorescence analysis of β-catenin in liver cancer cells with 10μmol/L shizukaol D treatment for 24 h. Red: staining for active β-catenin, blue: nuclear staining by DAPI. Upper: Focus cells; lower: SMMC-7721 cells. (E) Western blot analysis of the expression of endogenous Wnt target genes. SMMC-7721 cells were treated with 20μmol/L shizukaol D for 24 h before collectedand GAPDH was set as control. (F) mRNA expression level of endogenous Wnt target genes,c-myc, cyclin D, Tcf-1, LEF1, wnt3a and FGF18, by QPCR. Total RNA of SMMC-7721 cells was isolated after treated by 20μmol/L shizukaol D for 6 or 12 h, and then was transcripted reversely to cDNA. GAPDH was set as control. **: P<0.01. Values are presented as mean±S.D.

To explore the mechanism underlying the apoptosis, several signalling pathways were evaluated using luciferase reporter assays. Encouragingly, β-catenin/Tcf4 reporter activity, which was measured with reporter genes harbouring Tcf4-binding sites, largely decreased in response to shizukaol D treatment (Fig 4A). In 20–40% of HCC patients, β-catenin shows its nuclear accumulation and potential upregulated activity [20]. β-catenin is one of the hallmarks of Wnt/β-catenin pathway activation and the most straight forward method of inhibiting Wnt pathway is to target β-catenin by siRNA [21]. Interestingly, in our study, western blot analysis showed β-catenin levels were decreased in a dose- and time-dependent manner in both Focus (Fig 4B) and SMMC-7721 (Fig 4C) cells. This reduction in protein levels was further confirmed by immunofluorescence (Fig 4D). Furthermore, we detected several crucial mediators of Wnt signalling pathway and found the consistent down regulation of Dishevelled 2 (Dvl2) and Axin2 (Fig 4E). In addition, the phosphorylation of co-receptor LRP6 showed a reduction in protein levels. However, GSK-3β, a negative regulator which contributes to β-catenin degradation, did not change. Finally, we examined the Wnt signal pathway target genes by real-time PCR (qRT-PCR). C-Myc, Cyclin D, Tcf-1, LEF1, wnt3a and FGF18 were significantly repressed by shizukaol D, even at earlier time point (Fig 4F), confirming impeded β-catenin activity. Altogether, the results above demonstrated shizukaol D restrains cell survival through repressing Wnt/β-catenin pathway activity.

### Wnt pathway activation compensated for liver cancer cell viability

**Fig 5:**
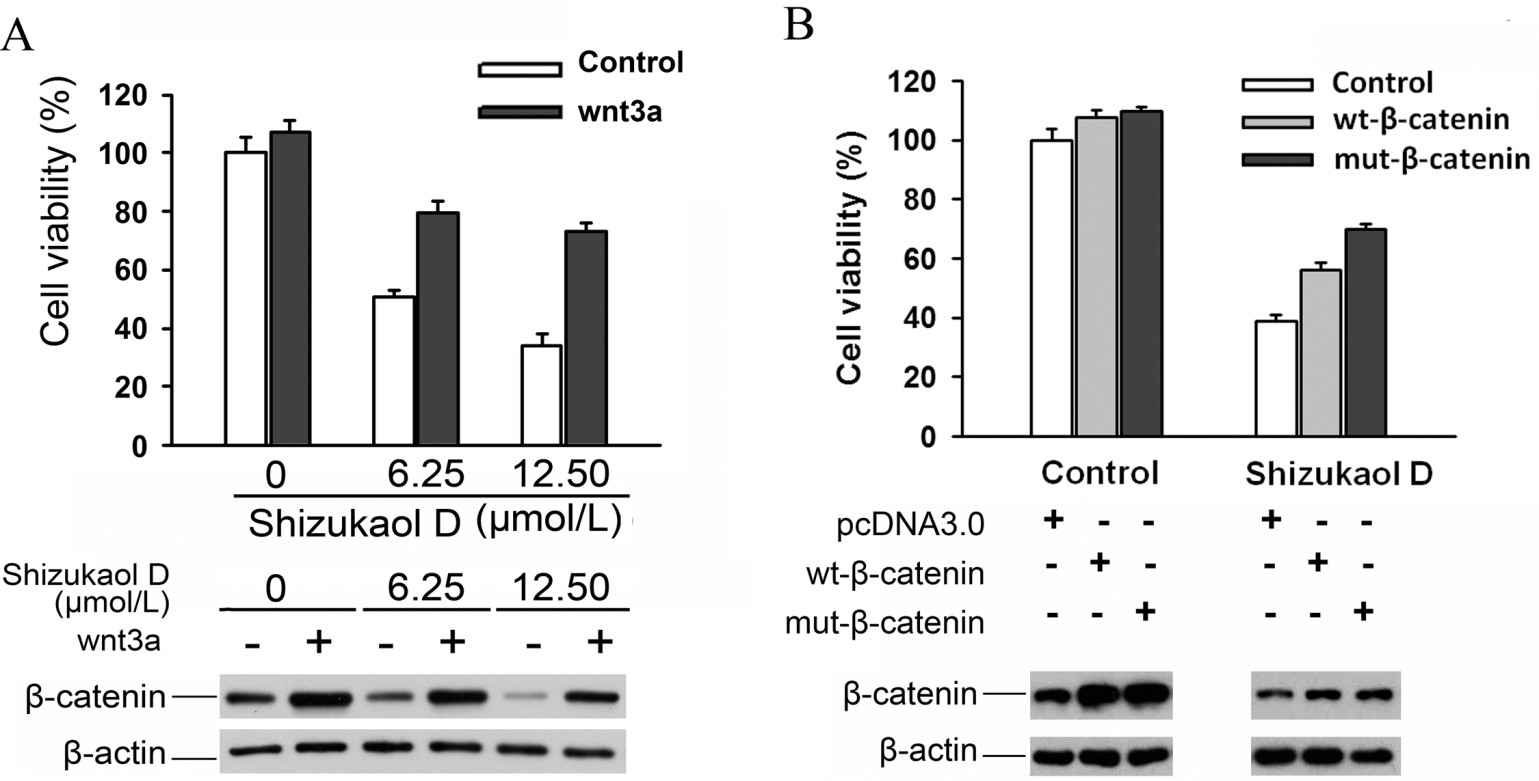
**Actviation of Wnt pathway rescued inhibitory effect of shizukaol D on liver cancer cells**. (A) The cell viability of SMMC-7721 cells treated by shizukaol D increased when incubated with wnt3a conditioned medium. (B) The cell viability of SMMC-7721 cells treated by shizukaol D increased when transfected with wt-β-catenin or mut-β-catenin. The expression of β-catenin protein of corresponding sample was analyzed by western blots and illustrated below. β-actin monoclonal antibody was used to confirm equivalent loading.

There are numerous ways to activate the Wnt pathway, such as adding wnt3a protein to cell culture media or increasing the expression of β-catenin. The viability of SMMC-7721 cells rebounded when they were cultured with wnt3a-conditioned media, regardless of whether they were simultaneously being incubated with 6.25 or 12.50 μmol/L of shizukaol D, although the cell viability did not reach the level of the control (Fig 5A). An alternative method of activating the Wnt pathway is via transfection with plasmid expressing β-catenin. After incubation in 12.5 μmol/L of shizukaol D for 48 hours, the viability of SMMC-7721 cells that had been transfected with plasmids expressing either wt-β-catenin or mut-β-catenin increased significantly compared to cells transfected with an empty carrier (plasmid pcDNA3.0). Western blot showed wt-β-catenin or mut-β-catenin transfection produced increased β-catenin proteins than control in SMMC-7721 cells (Fig 5B). Furthermore, the cells that were transfected with the mut-β-catenin plasmid exhibited slightly higher viability than those transfected with wt-β-catenin. These results suggest that Wnt pathway activation compensates for viability of liver cancer cells which were treated by shizukaol D.

## Discussion

Natural products often become sources of new drugs [22]. From the 1940s to the present, a total of 85, or 48.6%, of the therapeutic agents that have been approved by the FDA and similar organisations for the treatment of cancer were either natural products or derivatives of natural products [23]. Here, we showed that shizukaol D, a dimeric sesquiterpene isolated from *Chloranthus serratus*, induced growth inhibition in liver cancer cells. Only a single previous report has discussed the cytotoxicity of shizukaol D, in which cytotoxicity of extracts isolated from *Chloranthus japonicus* were evaluated, and the IC_50_ value of shizukaol D against SMMC-7721 was found to be 13.71±1.68 μmol/L [24]. In this study, the IC_50_ value of shizukaol D against SMMC-7721 was found to be 8.82±1.66μmol/L, which was close to the results of the previous report and proved the reliability of our methods.

Furthermore, we tested a series of liver cancer cells and found that the growth inhibition that induced by shizukaol D was both dose-dependent and time-dependent (Fig 2). Several liver cancer cell lines exhibited higher sensitivity to this compound, especially Focus cells. The inhibitory effect of shizukaol D on liver cancer cells was in accordance with the results of colony formation efficiency (Fig 2F and 2G).The growth inhibition that was caused by shizukaol D was significant compared to that caused by 5-FU, especially when concentrations exceeded 6.25 μmol/L (Fig 2B). 5-FU is a pyrimidine analogue that has been used to treat cancer patients; it has been administered systemically for the treatment of anal, breast, skin, oesophageal, stomach, pancreatic and colorectal cancers. Although the cytotoxicity that induced by shizukaol D was not robust at 24 h after treatment, it still exceeded that induced by 5-FU and therefore the optimisation of shizukaol D might serve as a promising direction to developing novel anti-tumour agents.

To uncover the mechanism underlying the observed growth inhibition, a luciferase reporter system was used to assess variance in different cell signal pathways that are involved in cell proliferation. The results indicated that shizukaol D attenuated Wnt pathway signalling (Fig 4A) in a manner similar to XAV939, which has been demonstrated to be a potent inhibitor of Wnt/β-catenin signalling [25]. The Wnt pathway is a key developmental pathway that is also involved in the pathogenesis of multiple types of cancer [26], and it presents a potential targeted pathway for therapeutic intervention in patients with HCC [27]. Next generation sequencing (NGS) has revealed that the Wnt signalling pathway is a key oncogenic driver in HCC patients [28]. The Wnt signalling pathway is also involved in apoptosis in cytopathic cells. In vivo, IFN-α2b treatment inhibits the Wnt/β-catenin/TCF pathway and promotes programmed cell death [29]. Two Wnt signalling pathway components, AXIN2 and FRA1, were found to exhibit decreased expression during hepatic stellate cell apoptosis [30]. In our study, shizukaol D increased the ratio of sub-G1 cells, which are products of apoptosis (Fig 3A), as well as the quantity of the 89kD cleaved PARP (Fig 3B), which is a substrate of the apoptosis-related protease caspase 3 [31]. The expressions of Wnt pathway target genes, such as C-myc, cyclin D, Tcf-1, LEF1, wnt3a, FGF18, were significantly repressed by shizukaol D (Fig 4E). These data suggested that cell death and apoptosis in liver cancer cells treated with shizukaol D were associated with attenuation of the Wnt pathway.

Moreover, western blotting revealed that the expression of β-catenin in liver cancer cells decreased in both a time- and dose-dependent manner (Fig 4B and 4C).The β-catenin protein, which is encoded by the CTNNB1 gene, plays an important role in canonical Wnt pathway signalling. In the absence of Wnt signalling, the cytosolic protein β-catenin is targeted and degraded by a multi-protein complex that includes axin1, APC, GSK3b and CK1 [32]. Activation of the Wnt signalling cascade disrupts the β-catenin degradation complex, then β-catenin proteins accumulate in the cytoplasm and translocate into the nucleus. Nuclear β-catenin acts as a transcription factor activating downstream target genes, such as c-myc, cyclin D1 [33, 34] and suvivin [35]. A decrease in β-catenin expression accompanied the liver cancer cell growth inhibition that was induced by shizukaol D treatment, which implied that growth inhibition resulted from modulation of the Wnt pathway.

To determine whether activation of the Wnt pathway prior to shizukaol D treatment rescued the observed effects, two different methods were employed in this study to activate the pathway: liver cancer cells were cultured in wnt3a-conditioned media or were alternatively transfected with plasmids encoding β-catenin. In both cases, cell viability increased after treatment with shizukaol D (Fig 5A and 5B). Cell viability following transfection with wt-β-catenin was slightly decreased compared to transfection with mut-β-catenin, which might be because S33A, S37A and T41A are GSK3 regulatory cassettes [36] and are vulnerable to shizukaol D. However, Wnt pathway activation did not offset the effects of shizukaol D in liver cancer cells fully and therefore additional mechanisms of growth inhibition may take effects at the same time. The rebound of viability in liver cancer cells that were treated with shizukaol D after activation of Wnt signalling verified that Wnt pathway is a potential target of shizukaol D and suppression of Wnt pathway by the compound leads to reduction of the proliferation and survival of liver cancer cell lines.

Natural compounds have been shown to be useful both individually and in combination with common therapies in preventing tumour progression and treating human malignancies [37]. Based on our findings, shizukaol D, a dimeric sesquiterpene isolated from *Chloranthus serratus*, might serve as a potential candidate for the treatment of liver cancer, although the identification of its direct target requires further investigation.

## Conclusions

In summary, our data demonstrated that shizukaol D exerted an inhibitory effect on the growth of liver cancer cells in a dose- and time-dependent manner, which was in part as a result of its modulation of the Wnt signalling pathway.

## Supporting Information

S1 Fig The mass spectrometryof shizukaol D. (TIF)

S2 Fig The 1H NMR spectra of shizukaol D. (TIF)

S3 Fig The HPLC of shizukaol D. (TIF)
