# Supplementary figures and images for: Shizukaol D, a Dimeric Sesquiterpene Isolated from Chloranthus serratus, Represses the Growth of Human Liver Cancer Cells by Modulating Wnt Signalling Pathway
Source: PLoS One. 2016 Mar 24;11(3):e0152012. doi: 10.1371/journal.pone.0152012 (PMC4807009; doi:10.1371/journal.pone.0152012)

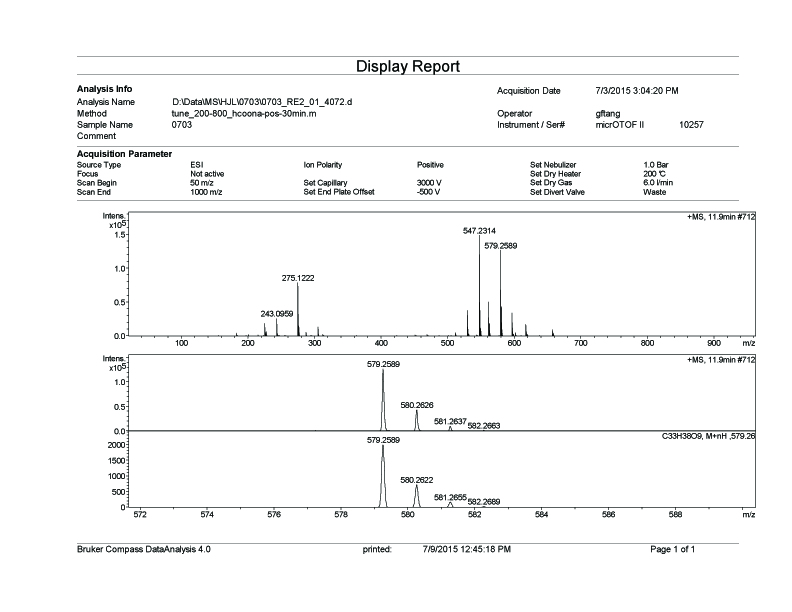

Supplement: S1 Fig — (TIF) [file pone.0152012.s001.tif]

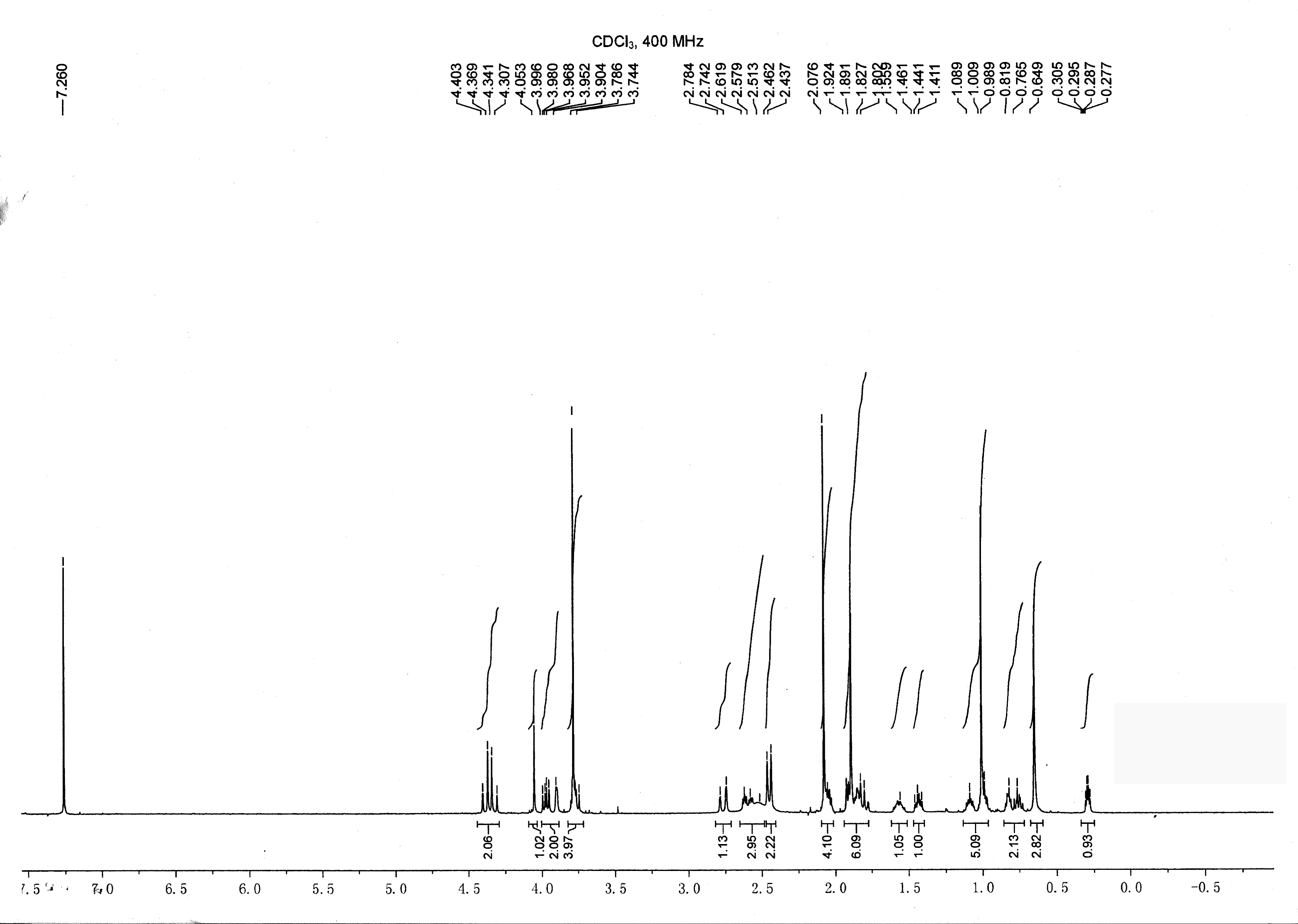

Supplement: S2 Fig — (TIF) [file pone.0152012.s002.tif]

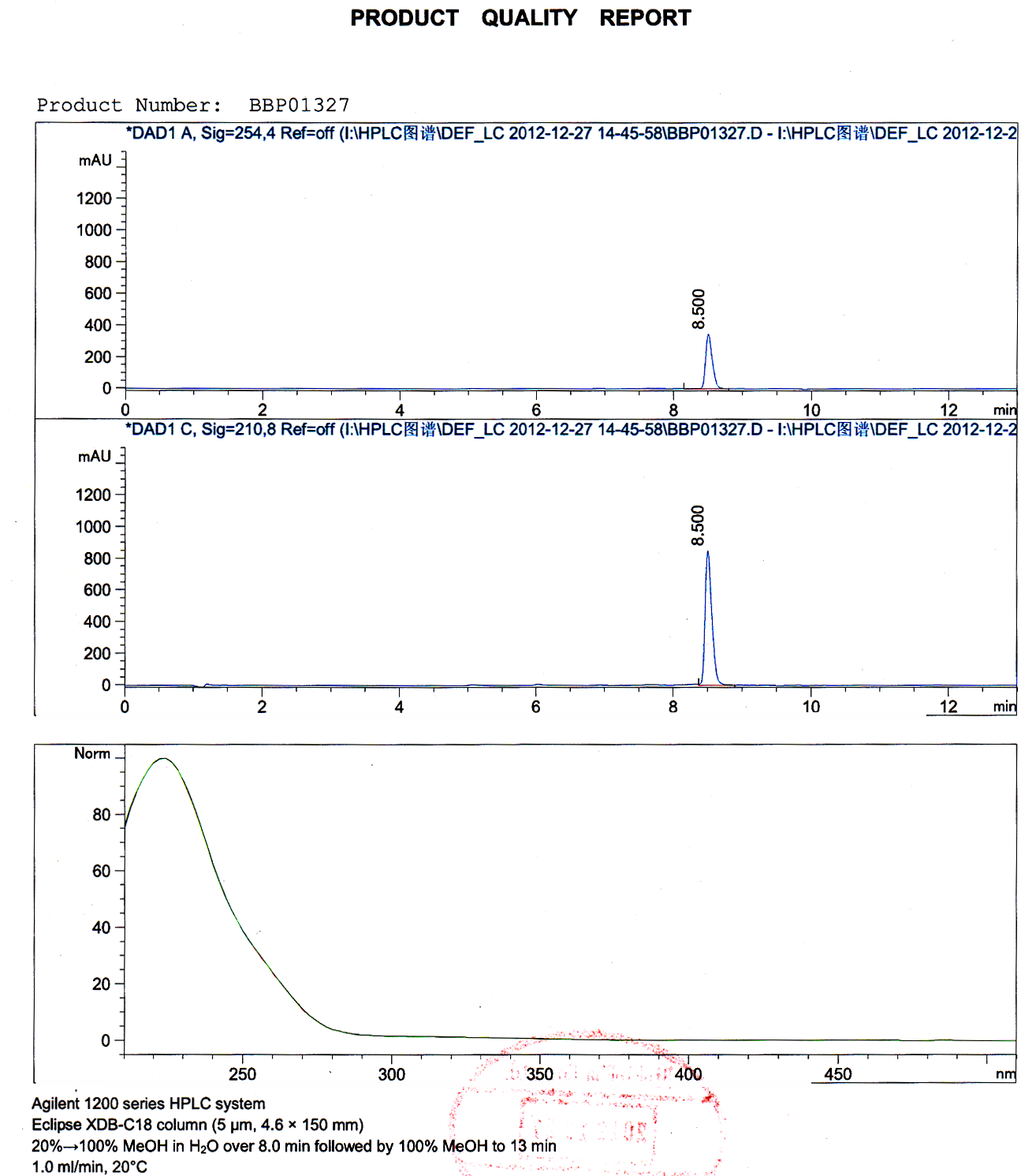

Supplement: S3 Fig — (TIF) [file pone.0152012.s003.tif]
